# Longitudinal analysis of large social networks: Estimating the effect of health traits on changes in friendship ties

**DOI:** 10.1002/sim.4190

**Published:** 2011-02-02

**Authors:** A James O'Malley, Nicholas A Christakis

**Affiliations:** a Department of Health Care Policy, Harvard Medical School180 Longwood Avenue, Boston, MA 02115, U.S.A.; b Department of Sociology, Harvard UniversityCambridge, MA 02140, U.S.A.

**Keywords:** dyadic independence, hierarchical model, homophily on health traits, longitudinal, social network

## Abstract

We develop novel mixed effects models to examine the role of health traits on the status of peoples' close friendship nominations in the Framingham Heart Study. The health traits considered are both mutable (body mass index (BMI), smoking, blood pressure, body proportion, muscularity, and depression) and, for comparison, basically immutable (height, birth order, personality type, only child, and handedness); and the traits have varying degrees of observability. We test the hypotheses that existing ties (i.e. close friendship nominations) are more likely to dissolve between people with dissimilar (mutable and observable) health traits whereas new ties are more likely to form between those with similar (mutable and observable) traits while controlling for persons' age, gender, geographic separation, and education. The mixed effects models contain random effects for both the nominator (ego) and nominated (alter) persons in a tie to account for the fact that people were involved in multiple relationships and contributed observations at multiple exams. Results for BMI support the hypotheses that people of similar BMI are less likely to dissolve existing ties and more likely to form ties, while smoker to non-smoker ties were the least likely to dissolve and smoker to smoker ties were the most likely to form. We also validated previously known findings regarding homophily on age and gender, and found evidence that homophily also depends upon geographic separation. Copyright © 2011 John Wiley & Sons, Ltd.

## 1. Introduction

An important question in social network analysis is whether an observed association in individual characteristics between connected individuals is a consequence of social influence (i.e. inter-personal forces that act once the tie is formed) or whether individuals who are similar (or dissimilar) are more likely to form (or dissolve) ties. The mechanisms are known as induction and homophily, respectively, with the latter commonly described as ‘birds of a feather flock together’. In most situations, it is likely that both effects occur simultaneously 1. However, distinguishing the effects is a particularly important problem, especially in social epidemiology where there is an interest in determining whether the actions of individuals to whom a person is connected affect an individual's health behavior?

Much prior work has documented the importance and ubiquity of homophily 2–5. When evaluating the role of induction (or peer effects) in any area, including health, it is important to ascertain the extent of homophily 6, 7. Distinguishing homophily from peer effects is facilitated by longitudinal data, yet, a general feature of many (but not all) prior studies of homophily is that they have used cross-sectional data and so have not examined the effect of individuals' traits on change in network ties (the work by Runger and Wasserman is an exception 8).

Our interest here is to use a long-term, longitudinally resolved, large network dataset to evaluate the magnitude of homophily along health-relevant dimensions. We focus on traits that are both apparent (i.e. observable to peers) and latent, and that are both mutable and immutable. We examine friendship ties in the Framingham Heart Study Social Network (FHS-Net) 9, 10 and in particular nominations of a ‘close friend’. While there is a plethora of reasons besides homophily that might cause the network (i.e. individuals' friendship status) to change—including changes in residence, personal conflicts or disputes, political affiliations, and other factors—we focus here on the role of homophily on a variety of measured attributes. In particular, we focus on the mutable health traits of body mass index (BMI), smoking, depression, blood pressure, body proportion, and muscularity and, for comparison, we also consider the four relatively immutable traits of height, personality type (type A versus type B), birth order, only child, and handedness (left-or right-handed). Personality type could perhaps vary over time but, as it was only collected at exam 2 in the FHS, it is immutable for our purposes. To minimize bias due to endogeneity (effects of uncontrolled factors on network evolution), we control for other observed variables (age, gender, living location, and education) that may be related to network evolution.

Our hypothesis is that nominations of close friendships are more likely to dissolve between actors who are dissimilar and conversely that new ties are more likely to arise between actors who are more similar in observable ways. Less observable traits like blood pressure provide sanity tests in the sense that we expect them to have no effect. We also conducted sensitivity analyses in which we evaluated whether ties are more likely to dissolve if actors' behavior is diverging and to form if actors' behavior is converging.

Our work is innovative in the following respects: (i) our dataset has special features, including a large sample size and very long follow-up with repeated observations; (ii) we are able to model both tie formation and tie dissolution in parallel models; (iii) we are able to account for whether the ties between pairs of individuals are reciprocated or not; and (iv) we are able to assess the impact of geographic separation on the process of homophily.

We emphasize that here we focus on the effect of health behaviors on network ties, rather than on the reverse issue of the effect of ones network ties on health. Prior work that analyzed the spread of attributes on the network took the network structure as given, and conditioned on the structure and on the individual attributes to look for inter-personal effects 9–11. That is, conditional on the existence of a tie, did the change in an attribute in one person (the ego), influence change in an attribute in the other person at the other end of the tie (the alter)? Here, we tackle the problem of whether attributes actually determine tie formation or dissolution. By looking at the ‘other side of the coin’, the present work augments previous work and motivates future work with this dataset (e.g. methods that fit the two problems simultaneously as described by Steglich *et al*. 1).

## 2. Methods

### 2.1. Data

We analyzed 32 years of data on individuals' characteristics obtained from the Framingham Heart Study (FHS), configured as a social network. To ascertain the network ties, we computerized information from archived, handwritten documents that had not previously been used for research purposes, namely, the administrative tracking sheets used and archived by the FHS by personnel responsible for calling participants in order to arrange their periodic health exams ever since the Offspring cohort of the FHS began in 1971.

The tracking sheets were used as a way of optimizing participant follow-up by asking participants to identify people close to them. But they also implicitly contain valuable social network information. These sheets recorded the answers when all 5124 members of the Offspring cohort were asked to identify friends, neighbors (based on address), coworkers (based on place of employment), and relatives who might be in a position to know where they (the egos) would be in two to four years. The key fact here that makes these administrative records so valuable for social network research is that, given the compact nature of the Framingham population in the period from 1971 to 2003, many of the nominated contacts were themselves also participants of one or another FHS cohort.

We have used these tracking sheets to develop friendship links for FHS Offspring participants to other participants in any of the four ongoing FHS cohorts; in addition to the Offspring cohort, these cohorts included the Original cohort, the Generation 3 cohort, and the OMNI cohort (for details, see Christakis and Fowler 9 and their Supplementary Appendix). The tracking sheets allow us to know which participants nominated or were nominated by others as a close friend at each exam 12–17. The status of friendship between two people (a dyad) is identified by each party identifying the other as a close friend or not, yielding four possible dyadic states (null, directional (either direction), and mutual). Named close friends can be in any of the four FHS cohorts.

The FHS *close friend* network is the network analyzed in this paper. Of the individuals named by offspring cohort members as close friends, 55 per cent are also participants in the FHS with the majority of these (68.5 per cent) in the offspring or subsequent cohorts. The total number of dyads that have non-null status in any exam is 2572; these involve 3754 unique actors.

The offspring cohort includes seven waves of health exams conducted approximately four years apart and such that the waves do not overlap (the time period of the exams was centered in 1973, 1981, 1985, 1989, 1992, 1997, and 1999). At each exam, a detailed medical assessment is performed yielding an extensive array of personal characteristics and medical information, including the individuals' height and weight, smoking status, blood pressure, evaluation of depression, and (at some exams) girth measurements (e.g. waist, hip, and arm girth), education, and handedness. We have also been able to correctly assign addresses to virtually all subjects at all the waves they came in for examination. We can thus compute distances between individuals 18.

### 2.2. Key variables

The dependent variable, denoted *Y*_*ijt*_, is the binary indicator of whether a tie exists from actor *i* to actor *j* at exam *t*. The predictors fall into three categories: network variables (e.g. whether or not a tie in the reverse direction exists), health traits (e.g. BMI, smoking), and other covariates (e.g. characteristics of the actors).

We include *Y*_*jit*_ as a predictor to control for reciprocity (also known as mutuality) or the effect of *j* naming *i* as their friend on the likelihood of *i* naming *j* as their friend. In this longitudinal setting, we are interested in whether being reciprocally named as a friend affects the likelihood of an existing tie dissolving or of a new tie forming.

The mutable and highly observable health traits of interest are: BMI, body proportion, muscularity, and smoking. Of these, muscularity is likely the least observable trait. Mutable but less observable traits are depression score (a scale variable about which a binary-valued clinical measure of depression is often defined) and blood pressure (a continuous measure). The immutable but more observable traits considered are height and personality type. The immutable and less observable traits are birth order, being an only child, and handedness.

Body proportion is defined herein as height/(waist girth), 19 muscularity as (arm girth)/(waist girth), birth order as (*n*_siblings_−*r*_order_)/*n*_siblings_ where *n*_siblings_ is the number of siblings including oneself and *r*_order_ is the rank from oldest (1) to youngest (*n*_siblings_) of an individual's age among their siblings, and personality type as the binary indicator of type A personality.

For continuously valued predictors (BMI, body proportion, muscularity, depression, blood pressure, height, and birth order), the dissimilarity of a variable *X* between individuals *i* and *j* at time *t* is defined as


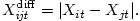


Two related predictors are whether the behavior is more pronounced in the ego (the individual naming) than the alter (the individual named)


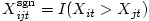


and the average value of the trait across the dyad


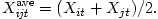


The vector containing the key predictors considered for continuous trait *X* is therefore *X*_*ijt*_ = (*X*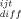, *X*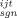, *X*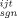 × *X*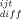, *X*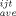)^T^.

**Figure 1:**
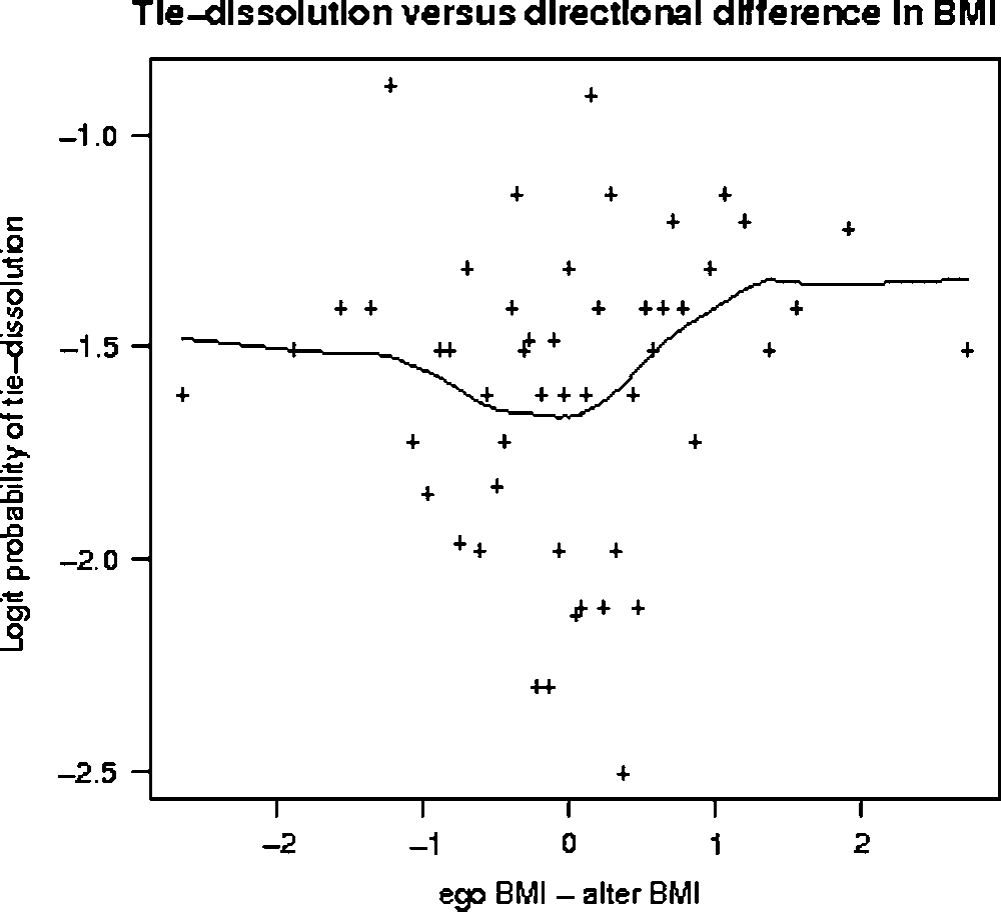
Scatterplot of logit of tie-dissolution proportions versus BMI difference. *Note*: Each data point corresponds to a ‘bin’ containing 2 per cent of the observations where bin membership is determined from the quantiles of ego BMI −alter BMI. The proportion of tie-dissolution events transformed to the logit scale is plotted against the mean BMI difference for the 50 bins.

**Figure 2:**
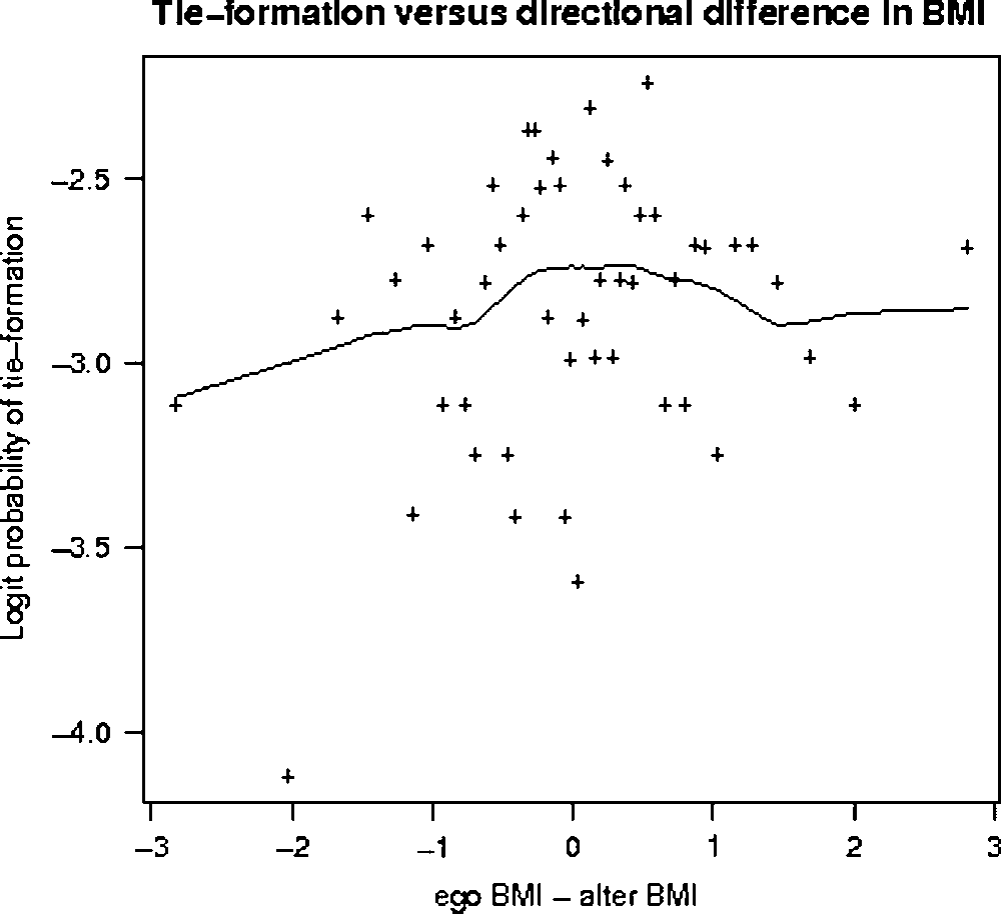
Scatterplot of logit of tie-formation proportions versus BMI difference. *Note*: Each data point corresponds to a ‘bin’ containing 2 per cent of the observations where bin membership is determined from the quantiles of ego BMI −alter BMI. The proportion of tie-formation events transformed to the logit scale is plotted against the mean BMI difference for the 50 bins.

In what follows, we justify the use of these variables to summarize the values of a continuous trait across the ego and the alter. A variable *X* may be expressed as *X* = sgn(*X*)|*X*|, where |*X*| denotes the absolute difference and sgn(X) equals 1 if *X*>0 and −1 otherwise. A nice feature of decomposing *X* into |*X*| and sgn(X) is that they represent the main effects of magnitude and directionality and their product is the associated interaction. Scatterplots of the logit-transformed proportions of ties broken and new ties formed, respectively, versus the directional difference of BMI (ego–alter), grouped in small subintervals, are displayed in Figures 1 and 2. Over the interior region of the plot, the trend estimated using a lowess smoother is approximated by a V for tie dissolution and by an inverted-V for tie formation suggesting that the absolute difference is an appropriate dissimilarity metric for continuous traits 20. The absolute value has been used by others as a metric of dissimilarity of continuously valued variables. For example, Zeng and Xie use the absolute difference to test for homophily in age, grade-point average, and socio-economic status 20. We also considered using other distance metrics, such as the squared distance, but none offered general improvement over |*X*|.

For binary-valued traits (smoking, personality type, only child, and handedness), the key predictors are the indicator variables:


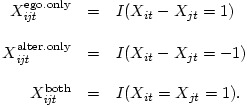


These variables indicate whether the ego, the alter, or both the ego and alter exhibit the trait (the left out category is that neither ego nor alter exhibits the trait), respectively. *X*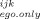 and *X*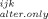 are dissimilarity measures whereas *X*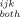 is a measurement of prevalence. The vector of key predictors for binary trait *X* is, therefore, *X*_*ijt*_ = (*X*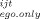, *X*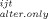, *X*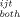)^T^.

Other characteristics are contained in the vector *Z*_*ijt*_. We control for age (the absolute value of the difference in age and average age), gender (both male and both female indicators), geographic separation (the physical geodesic distance between persons' residential abodes or the absolute change in this over the current exam and the preceding exam), and education (absolute difference and average of an ordinal variable ranging from 0 (none) to 8 (post graduate)). We see age and gender (and education) as being relevant in and of themselves, but, also to proxy for other traits, including enculturation regarding social behavior, wealth, and so on. Our main objective here was to include them as control variables for the other effects that are our explicit focus. We scaled all elements of *X*_*ijt*_ and *Z*_*ijt*_ to have mean 0 and standard deviation 1, allowing effects to be directly compared between predictors.

Although the patients are examined on different days and have different lengths of time between exams, the exact date when the status of a friendship nomination would have changed is unknown. Therefore, we treat the data as if everyone was examined at the same time at each exam, yielding a regular longitudinal dataset.

### 2.3. Missing data

For each member of the Offspring cohort, we have data from up to seven medical examinations. Only 24 individuals dropped out of the offspring cohort (0.47 per cent). However, on occasion, individuals miss an exam or the data recorded from an exam is only partially complete. The latter occurs most frequently when a variable is purposely not included in an exam and on a small number of cases is missing due to chance (e.g. due to a data recording error); these data are most likely missing completely at random.

We treated height, birth order, and only child as time invariant as almost all study participants are adults. There were 10 cases where handedness apparently changed from left to right or vice versa; we treated these as if the patient was left-handed throughout making this trait ‘ever left-handed’.

Tie exams in which an individual misses an exam, dies, or otherwise terminates involvement with the FHS are excluded from the analysis. Thus, we implicitly assume that earlier observations on subjects who die during the study period adhere to the same underlying data generating process as observations on individuals who survive the entire study period.

### 2.4. Statistical analysis

The goal of the analysis is to determine the effect of actors' health traits on the status of close friend nominations in the FHS network. We propose a stochastic model in which the transition probabilities that the tie changes from connected to unconnected or vice versa depend on the status of the tie at the preceding exam and characteristics of the individuals.

The Markov property—namely that the status of a tie at the next exam depends only on the current status of the dyad (the pair of ties in each direction)—is inherent to the model. One of the reasons we expect the Markov property to hold is that the observation times are at least two years apart and so it is unlikely that the status of a dyad two or more exams in the past exerts much influence given the status at the preceding exam. Thus, conditional on *Y*_*ijt*−1_ and *Y*_*jit*−1_, dyadic status at exam *t*−2 or earlier is considered uninformative.

Because participants generally name a single close friend contact at each exam, the observed network is sparse (as shown in Table I (A, B), a few named more than one contact, 0.5 per cent or less). Owing to its sparseness, cross sections of the FHS friendship network are not amenable to analyzing the effects of higher order effects such as transitivity 21. Therefore, we only consider models that, conditional on individual random effects, exhibit dyadic independence at each exam. An advantage of such parsimony is that the network for a random sample of dyads has the same distribution as the whole network; thus the model has the attractive property of being generative. However, because the data are rich longitudinally due to almost non-existent study attrition, the design still allows valuable information about the effect of health behaviors on dyad status to be obtained and presents a unique opportunity to study the dynamic properties of the network at the level of the dyad. The innovative feature of our models is thus the longitudinal component, which is very unusual for network models.

**Table I:** Degree distribution by exam: (A) for the offspring cohort members and (B) for those offspring cohort members who were in a non-null dyad at some point

	Exam number						
	
Number of named friends	1	2	3	4	5	6	7
(A)							
0^*^	0.766	0.760	0.756	0.755	0.762	0.771	0.784
1	0.233	0.239	0.242	0.243	0.237	0.228	0.214
2	0.002	0.002	0.002	0.002	0.001	0.001	0.001
Average^Degree^	0.236	0.242	0.246	0.247	0.238	0.230	0.217
(B)							
0^*^	0.357	0.339	0.331	0.327	0.348	0.374	0.407
1	0.639	0.656	0.663	0.668	0.650	0.624	0.589
2	0.004	0.005	0.005	0.005	0.002	0.003	0.004
Average^Degree^	0.647	0.666	0.674	0.678	0.654	0.629	0.596

*Note*: ^*^Individuals who missed exam.

In recognition of the limited cross-sectional capacity of the data, we fit separate models for the tie-dissolution and tie-formation probabilities. The binary regression models for the tie-dissolution and tie-formation probabilities are given by


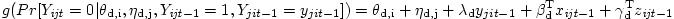
(1)

and


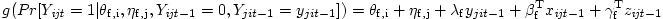
(2)

where θ_d, i_∼*N*(0, σ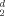), η_d, j_∼*N*(0, τ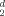), θ_f, i_∼*N*(0, σ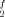), and η_f, j_∼*N*(0, τ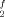) are the sender (nominator) and receiver (nominated) random effects, respectively. We analyze the data using the logit link *g*(*p*) = log(*p*/(1−*p*)). The fixed-effect parameters λ = (λ_d_, λ_f_)^T^, β = (β_d_, β_f_)^T^, and γ = (γ_d_, γ_f_)^T^ quantify the effects of reciprocity, the health behavior variables, and the other covariates on the probability of a given tie dissolving (equation (1)) or forming (equation (2)), respectively. The models given by (1) and (2) are Markov conditional on the latent variables (i.e. random effects) for actors' sender and receiver effects and so the probability of tie transitions depend only on the current state of the tie and actor-specific latent variables. The models are thus a hierarchical generalized linear model with a bivariate random effect and lagged outcomes to account for cross-sectional and longitudinal dependence. While dyadic independence is a strong assumption, it is made more believable by the fact that the assumption is conditional on observed and unobserved actor-level attributes and associated effects 21. We compared the models presented in this paper to those with random effects for each dyad but found that they did not improve upon the bivariate actor-level random effect specifications in (1) and (2).

Because θ_*i*_ = (θ_d, i_, θ_f, i_)^T^ and η_*j*_ = (η_d, j_, η_f, j_)^T^ are random, inferences about β, λ, and γ are based on variation both between and within ties such that the weight of the former decreases as τ^2^ increases. We are interested in the effects of the predictors on individuals' close friend choices, as opposed to population average effects, so inferences focus on the parameters themselves rather than on marginal effects that average over observed (*z*_*ijt*_) and unobserved (θ, η) variables.

The predictors are lagged to ensure that β is the effect of differences in health traits between individuals on their friendship status and not the reverse. Consequently, data from the first exam are only used to form predictors and do not contribute records used in model fitting. To estimate the effect of changes in health traits on tie dissolution and tie formation, a sensitivity analysis in which both *x*_*ijt*−1_ and *x*_*ijt*_ are included in the model was performed.

If not for the situation when a study participant nominates multiple persons (the interviewer has no prerogative to pick one over another and so records all nominations), the sender variance components (σ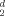, σ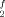) would be pure measures of unobserved heterogeneity between exams in the propensity of an individual to dissolve or form a tie. The receiver variance components (τ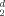, τ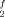) quantify unobserved heterogeneity between individuals within and across exams. We refrained from fitting a hierarchical extension of the p2 model 22, 23, a traditional model for dyadic data, for two reasons. First, because the FHS only requires that participants name a single close friend at each exam, (σ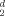, σ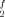) are expected to be very small relative to (τ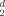, τ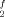) and possibly close to 0, making the correlation between sender and receiver effects, a key parameter of the p2 model, difficult to estimate. Second, the separable tie-dissolution/tie-formation model in (1) and (2) is easier to fit (especially on a network the size of ours) 24, 25.

Initially, we fit separate models for each trait. Thus, in the first model *x*_*ijt*−1_ contained the predictors summarizing the actors' BMI; a separate model evaluates the predictors of body proportion, muscularity, and so on through the remaining traits of interest (depression, blood pressure, height, smoking, personality type, birth order, only child, handedness). This allows the marginal effect of each health trait to be determined. We then tested whether controlling for other traits modified the effect(s) of the trait being analyzed. However, with the exception of BMI and smoking, and BMI and body proportion, the traits had very little impact on each other, implying that modeling a single trait at a time was sufficient. In particular, we were concerned that the effects of BMI and smoking may not be fully revealed if they are not inferred from a single model as it is well known that smoking can be a form of weight control 26. Indeed, the joint inclusion of BMI and smoking increased the magnitude of the BMI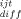 tie-formation and the smoking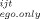 tie-dissolution effects, the latter becoming significant at the 0.05 level (Section 3.2). The correlations between the estimated coefficients of the BMI and smoking variables are (surprisingly) small, ranging from −0.033 (BMI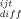 and Smoke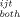) to 0.083 (BMI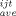 and Smoke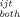). It makes sense that the largest and most positive correlation should occur between BMI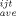 and Smoke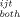 as these predictors are both measures of prevalence.

In a second sensitivity analysis, we substituted *y*_*jit*−1_ with three predictors: *y*_*jit*−1_*y*_*jit*_, (1−*y*_*jit*−1_)*y*_*jit*_, and *y*_*jit*−1_(1−*y*_*jit*_). These form a dynamic representation of reciprocity with specific effects for whether the incoming tie existed at the exams *t*−1 and *t*, whether the incoming tie was formed between exams *t*−1 and *t*, and whether the incoming tie dissolved between exams *t*−1 and *t*. The baseline level is non-existence of the tie at both exams. We performed this analysis to determine if the change in the status of the incoming tie had an effect on the change in status of the outgoing tie.

We used the lmer package in R 27 to fit the cross-classified random effect logistic regression models in (1) and (2). R was also used for data manipulation, supporting calculations, and figure development. All *p*-values reported in this paper assume two-tailed tests.

### 2.5. Sampling non-connected ties

Across the seven exams, a total of 1286 dyads (2572 potential ties) involving 1876 unique actors ever had a status other than null (no ties). Therefore, without considering the FHS members who did not name a FHS participant or who were not named by a FHS participant in the offspring cohort, there are approximately 1286 × 1285/2−1286 = 824969 dyads that remained null across the seven exams.

In the tie-formation analysis, we condition on dyads with null outbound ties at an exam and model the probability that the outbound tie forms (i.e. the given receiver is named) by the next exam. Given that the unit of response is the dyad exam, there are approximately 21 million ties that could form if each of 1876 actors knew of and could name every actor at exams 2 through 7, whereas a mere 568 ties transition from null to connected at some point (Section 3.2). To make the tie-formation model less computationally burdensome to fit, we randomly sampled dyads whose ties were unconnected at every exam (i.e. for whom a tie never formed) and combined these with the 1286 dyads that ever had a non-null tie. Specifically, for a randomly selected actor in each of these 1286 non-null dyads, we randomly sampled *k* actors for whom the resulting dyad was null across the seven exams, yielding an additional 1286*k* dyads (2572*k* tie-level observations). Such dyadic partners were restricted to offspring members who at some point nominated another FHS study participant to maximize the chance that the individuals actually know, and could have nominated each other. Dyads of individuals known to be relatives were excluded prior to sampling.

The ensuring analysis seeks to find the regression parameters that best discriminate between dyad exams where new outbound ties form and those where no tie forms. Although, the model in (2) is still appropriate for use, as for case–control analyses the intercept parameter does not reflect the overall proportion of null to non-null dyadic transitions.

The total number of dyads in the dataset for the tie-formation analysis is 1286(*k*+ 1), where *k* is the ratio of ‘controls’ to ‘cases’. We used *k* = 5 to compute the results reported here; larger values of *k* had little impact on the results (in general offering only slightly more precision). The sample size for the tie-formation analysis is the number of dyad exams at which the outbound tie could have transitioned from null to connected across the seven exams. The addition of the 6430 always-null dyads adds additional 38 580 (77 160) potential dyadic (tie)-level transitions across the seven waves to the tie-formation analyses. However, as noted in Section 3.2, some of these observations are not used due to missing dyadic status at the preceding exam.

## 3. Results

We first describe the set of 2572 dyads for whom at least a directional tie existed at some point during the study period. We describe the status of these dyads in terms of their degree distributions, density, and the extent to which their status changes.

Table I (A, B) shows the degree distribution across the seven exams for the full offspring cohort and the subset of individuals involved in non-null dyads at some exam respectively. By definition, the only difference in the distributions is the number of individuals with degree 0, which occurs when an individual names a non-FHS participant. When all offspring cohort members are considered about 70 per cent have degree 0 whereas 55–61 per cent of individuals in the non-null subset have degree 1 while in both cases only a small fraction (0.5 per cent or less) have degree 2 at any given exam. In both cases, the trend of the mean degree across exams is slightly concave with the maximum at exam 4, closely followed by the mean degree at exam 3.

The amount of information about the dynamic behavior of the network is a function of the number of observed transitions. The number of ties that changed status between exams 1 and 2, 2 and 3, 3 and 4, 4 and 5, 5 and 6, and 6 and 7 is 218, 261, 319, 187, 152, and 94, respectively. The total number of transitions between consecutive exams is 1231. An additional 149 transitions took place between exams separated by one or more exams with missing tie status (yielding a total of 1380 transitions).

Table II contains the counts of the number of transitions in dyad status over consecutive exams between the three states: null (no friendships), directional (name friend or named as friend but not both), and mutual (name and named as friend). The diagonal elements give the number of dyads with unchanged status from one exam to the next. The six dyads for whom *Y*_*ijt*_ = 1−*Y*_*jit*−1_ and *Y*_*jit*_ = 1−*Y*_*ijt*−1_ (i.e. a dyad with a directional tie that changes direction) are subsumed in the 2203 directional–directional transitions. The total number of dyads with outbound ties that dissolve before the next exam is thus: 553 + 6 + 10 + 79 = 648, whereas the number of dyads with inbound ties that form before the next exam is 505 + 5 + 6 + 52 = 568. The number of potential changes in dyad status that could occur is 6274 and so the percentage of dyads that change status is 1210/6274 = 19.29 per cent.

**Table II:** The number of transitions of each type at the dyad level

Exam_*t*−1_\Exam_*t*_	Null	Directional	Mutual
Null	2520	505	5
Directional	553	2203	52
Mutual	10	79	347

*Note*: ^*^Individuals who missed exam.

### 3.1. Evaluation of model suitability and results for control predictors

**Figure 3:**
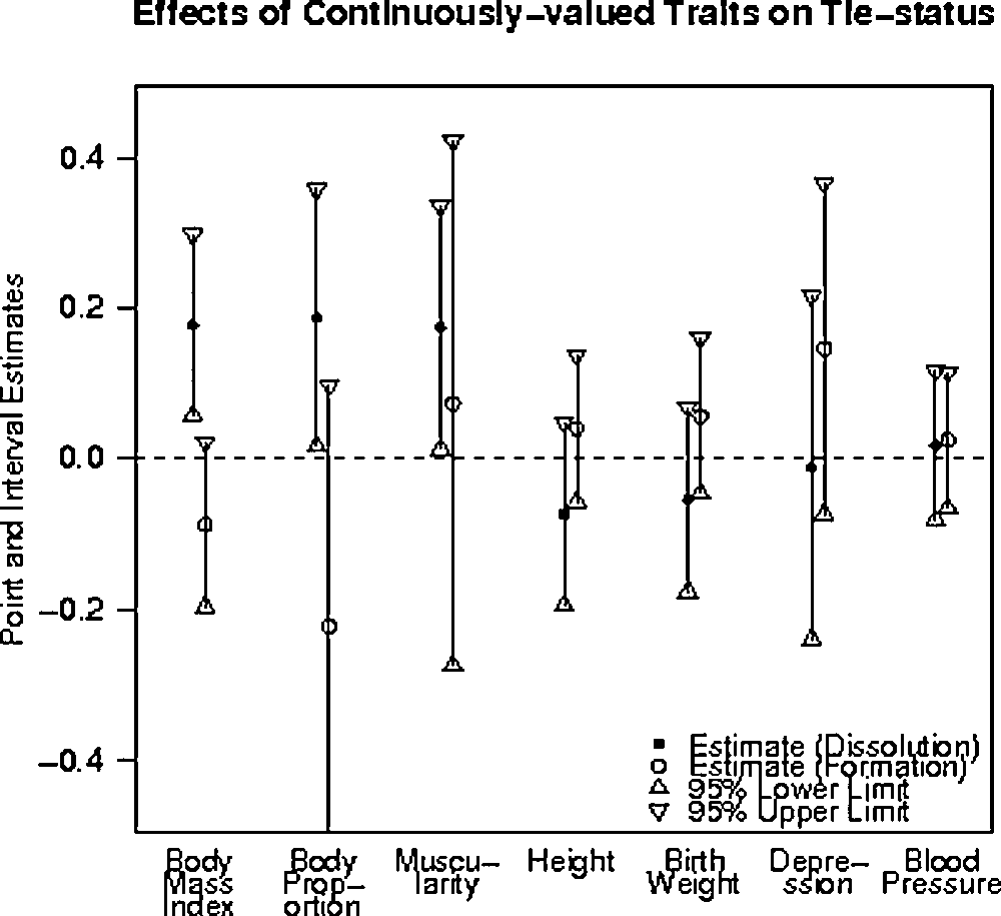
Summary results for continuously valued traits controlling for exam-number, lag inbound tie status, age difference, age average, both male, and both female. *Note*: From left to right the traits are in decreasing order of the sum of the absolute values of their effects in the tie-dissolution and tie-formation models.

The results of the fitted models are presented by first showing the full models when all of the elements of BMI_*ijt*−1_ = (BMI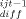, BMI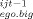, BMI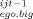 × BMI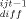, BMI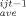)^T^ are in the BMI-Smoking models (Table III), and then showing the key results of the analyses for each of the traits in a separate table (Table IV) and figure (Figure 3). The suitability of the base model and all of the key substantive results can be presented in such a concise manner as the control predictors are the same in each model and their effects are largely invariant across traits (ruling out excessive collinearity with any of the key predictors).

**Table III:** Fitted model justifying use of (absolute) difference as key predictor for BMI (and other continuous traits)

		Tie dissolution				Tie formation			
			
Predictor type	Term	Estimate	SE	*z*-statistic	*p*-value	Estimate	SE	*z*-statistic	*p*-value
*Key predictors*									
	Lag Behavior: BMI Difference	**1.714**	**0.597**	**2.873**	**0.004**	−0.896	0.545	−1.643	0.100
	Lag Behavior: BMI Ego bigger	0.025	0.079	0.310	0.756	−0.036	0.069	−0.530	0.596
	Lag Behavior: BMI Directional Difference	0.003	0.013	0.195	0.846	0.018	0.012	1.450	0.147
	Lag Behavior: BMI Average	−**1.296**	**0.620**	−**2.091**	**0.037**	−0.142	0.543	−0.262	0.793
	Lag Behavior: Ego Only Smokes	−**0.328**	**0.154**	−**2.121**	**0.034**	0.019	0.127	0.154	0.878
	Lag Behavior: Alter Only Smokes	−0.036	0.157	−0.231	0.817	−0.183	0.134	−1.371	0.170
	Lag Behavior: Both Smoke	0.137	0.174	0.787	0.431	0.265	0.149	1.775	0.076
*Control predictors*									
	(Intercept)	−1.889	0.377	−5.007	0.000	−2.759	0.292	−9.461	<2e −16
	factor(exam)3	0.559	0.170	3.282	0.001	0.344	0.143	2.402	0.016
	factor(exam)4	0.902	0.161	5.603	0.000	0.639	0.136	4.706	0.000
	factor(exam)5	0.603	0.171	3.520	0.000	−0.230	0.166	−1.389	0.165
	factor(exam)6	0.232	0.198	1.171	0.242	−0.197	0.170	−1.157	0.247
	factor(exam)7	0.080	0.213	0.375	0.708	−0.589	0.204	−2.884	0.004
	Lag Inbound Tie	−0.480	0.141	−3.398	0.001	−1.349	0.148	−9.115	<2e −16
	Age Difference	0.040	0.008	5.045	0.000	−0.043	0.008	−5.657	0.000
	Age Average	0.006	0.006	1.001	0.317	0.007	0.006	1.348	0.178
	Both Male	−0.172	0.207	−0.829	0.407	0.330	0.138	2.384	0.017
	Both Female	−0.383	0.205	−1.867	0.062	0.306	0.136	2.250	0.024
	Lag Geographic Separation*	0.033	0.104	0.321	0.748	−0.041	0.075	−0.547	0.584
	Education Difference†	−0.035	0.070	−0.494	0.621	−0.033	0.069	−0.487	0.626
	Education Average†	−0.000	0.067	−0.007	0.994	−0.058	0.074	−0.776	0.438
*Standard deviations*									
	SD (ego)	0.000	0.000	NA	NA	0.000	0.000	NA	NA
	SD (alter)	0.583	0.732	NA	NA	0.686	0.894	NA	NA

*Note*:

* Only available for offspring cohort members.

† Only obtained at exam 3. Preliminary analyses indicated that BMI and smoking traits should be inferred simultaneously (Section 2.4). The non-significance of the geographic separation and the education variables led them to be dropped and the model refitted (estimates for other terms are from the refitted model). Bold font is used to highlight effects with *p*-values (based on two-tailed tests) less than 0.05.

**Table IV:** Summary results for the tie-dissolution and tie-formation analyses using hierarchical logistic regression when analyzing a single trait at a time (controlling for exam number, lag inbound tie status, age difference, age average, both male and both female)

	Tie dissolution				Tie formation			
		
Variable	Estimate	SE	*z*-statistic	*p*-value	Estimate	SE	*z*-statistic	*p*-value
BMI	**0.177**	**0.061**	**2.884**	**0.004**	−0.087	0.056	−1.563	0.118
Smoking								
ego only	−**0.071**	**0.033**	−**2.121**	**0.034**	0.003	0.027	0.093	0.926
alter only	−0.007	0.034	−0.194	0.846	−0.038	0.029	−1.323	0.186
both	0.022	0.026	0.832	0.405	0.040	0.023	1.752	0.080
Body proportion	**0.187**	**0.087**	**2.149**	**0.032**	−0.223	0.163	−1.362	0.173
Muscularity	**0.175**	**0.082**	**2.129**	**0.033**	0.074	0.178	0.414	0.679
Depression score	−0.012	0.116	−0.107	0.915	0.153	0.134	1.139	0.255
Blood pressure	0.018	0.051	0.354	0.723	0.025	0.046	0.533	0.594
Height	−0.074	0.062	−1.182	0.237	0.041	0.050	0.816	0.415
Personality type								
ego only	−0.033	0.044	−0.762	0.446	−0.004	0.036	−0.113	0.910
alter only	−0.059	0.044	−1.321	0.186	−0.001	0.036	−0.039	0.969
both	−0.048	0.051	−0.939	0.348	−0.006	0.041	−0.136	0.892
Birth order	−0.054	0.062	−0.872	0.383	0.057	0.053	1.082	0.279
Only child								
ego only	−0.024	0.032	−0.734	0.463	0.043	0.026	1.630	0.103
alter only	0.037	0.035	1.072	0.284	−0.031	0.029	−1.065	0.287
both	−0.016	0.027	−0.594	0.553	0.001	0.023	0.028	0.978
Handedness								
ego only	−0.005	0.032	−0.153	0.879	−0.003	0.026	−0.102	0.919
alter only	0.034	0.033	1.030	0.303	−0.038	0.029	−1.333	0.183
both	0.010	0.023	0.410	0.682	−0.016	0.027	−0.595	0.552

*Note*: Bold font is used to highlight effects with *p*-values (based on two-tailed tests) less than 0.05. The first through fourth segments of the table contain the mutable and more observable, mutable and less observable, immutable and more observable, and immutable and less observable predictors, respectively. Because BMI and smoking are analyzed in the same model, a total of 20 models are fit.

In the tie-dissolution (BMI-Smoking) model, BMI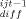 and BMI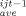 have highly significant effects whereas BMI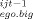 and BMI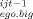 × BMI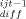 have minimal effects (Table III) over and above those of the other predictors. The lack of significance of the effects involving BMI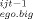 suggests that only the magnitude of the difference in BMI matters, and not whether ego BMI is bigger or smaller than alter BMI. BMI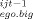 and BMI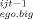 × BMI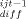 also have non-significant effects in the tie-formation model. Thus, there appears little justification for retaining BMI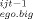 or BMI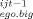 × BMI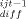. In additional analyses (not reported), we checked whether *X*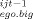 and *X*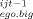 × X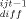 had significant effects for any of the other continuously valued traits. None were found. Therefore, we conclude that *X*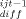 and *X*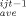 capture the full effect of the continuous traits in the analyses. The positive coefficient of BMI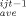 implies that individuals with a high average BMI between them are in general less likely to dissolve ties. However, there was no effect of BMI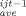 on tie formation.

In the tie-dissolution model, the presence of the reciprocated nomination (i.e. *Y*_*jit*−1_ = 1) has a strong negative effect implying that an outbound nomination was less likely to change if the dyad was mutual (i.e. the ego was named as a friend at the last exam by the alter). That is, individuals who name each other are less likely to change their nomination. When we replaced the inbound tie variable with the three-component dynamic reciprocity effect, we found that if the inbound tie had just formed (largest effect) or if the inbound tie had been in place for at least two exams, the outbound tie was much less likely to dissolve. However, if the inbound tie had just dissolved, the outbound tie was more likely to dissolve.

Surprisingly, the presence of an inbound tie at the previous exam was a deterrent against a new tie forming. We suspect that this result is an artifact of the FHS study instruction that participants name a close friend as their non-family contact, which generally resulted in a single friend being nominated at each exam. We suspect that the most popular individuals (i.e. those named by others) have the most stable friendships; they both attract nominations and have no reason to change their own close-friend nomination(s). Furthermore, the sensitivity analysis involving the three-component dynamic reciprocity effect revealed that although the effect of the inbound tie having just dissolved had a very large negative effect (around −1.7), the existence of an inbound tie for two or more exams and the indicator of a newly formed inbound tie also had negative effects (albeit of lower magnitude). Our interpretation of this result is that if an individual is thought so highly to be named at repeated exams by another individual, it is likely that they would have named them previously if they were ever going to.

The absolute difference of individuals' age, Age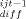, was a strong predictor in the tie-dissolution (negative effect) and tie-formation (positive effect) models, implying that homophily on age is an important factor in the dynamic behavior of the network. These results are consistent with the existing literature 4. Despite strong evidence (*p*-values <0.05 for both genders) that homophily on gender increased the likelihood of tie formation, there was little evidence that it protected against tie dissolution. The parameter estimate for lag geographic separation was positive for tie dissolution and negative for tie formation but neither effect was statistically significant (*p* = 0.748 and *p* = 0.584, respectively). Inclusion of the potentially endogenous variable, absolute change in the geographic separation between the current exam and the preceding exam had minimal effect on the other effects in the model, implying that friendship termination due to people moving home or out of Framingham did not confound the results. Finally, education was not significant.

The random effect standard deviations of the receiver effects in the BMI-Smoking models were 0.583 and 0.686 for the tie-dissolution and tie-formation processes, respectively, whereas as hypothesized the sender effects were close to 0.

All results for the tie-formation analyses were robust to different random samples of always-null dyads implying *k* = 5 is sufficient; because only non-null ties are conditioned on, the results for the tie-breaking analysis are not affected by re-sampling the always-null dyads.

### 3.2. Results for key predictors

After omitting dyad exams in which the status of the outgoing tie at the previous exam was not-nominated or missing, there were 4031 dyad exams eligible to transition from nominated to dissolved. Analogously, after omitting dyad exams in which the status of the outgoing tie at the previous exam was nominated or missing, there were 86 677 dyad exams eligible to transition from not-nominated to nominated. Because of occasional missing health traits, the exact number of observations used to fit each model varied with the trait analyzed.

**Figure 4:**
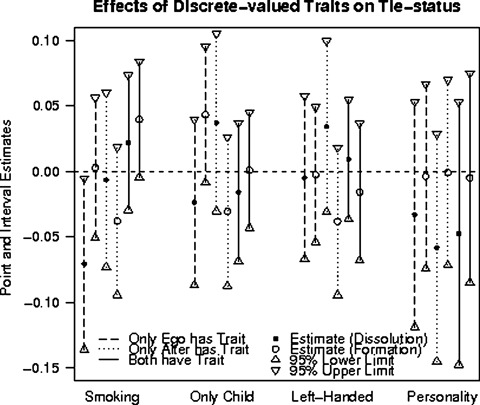
Summary results for discrete-valued traits controlling for exam number, lag inbound tie status, age difference, age average, both male, and both female. *Note*: From left to right the traits are in decreasing order of the total sum of the absolute values of their effects (only ego has trait, only alter has trait, both have trait) in the tie-dissolution and tie-formation models.

BMI, body proportion, muscularity, and smoking, the mutable and highly observable predictors yield several significant (*p*-value less than 0.05) results (Table IV and Figures 3 and 4). Of particular note, BMI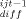 has a significant positive effect (estimate 0.177, *p*-value 0.004) on tie dissolution (implying individuals with similar BMI are less likely to change their nomination than those with disparate BMI at the preceding exam) and a negative but not significant effect (estimate −0.187, *p*-value 0.118) on tie formation. Because BMI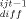 is the only key variable for which the 95 per cent confidence intervals in the tie-dissolution and tie-formation analyses do not overlap (Figures 3 and 4) it is the health trait for which the effect of homophily is the greatest. In general, traits whose effects of tie dissolution and tie formation have 95 per cent confidence intervals that do not overlap much are considered stronger determinants (in terms of statistical significance) of changes in dyadic status than traits whose 95 per cent confidence intervals have substantial overlap. In this regard, Figures 3 and 4 provide a joint statement across the tie-dissolution and tie-formation analyses, providing overall strong evidence that BMI similarity is protective of tie dissolution and an encouragement to tie formation.

Body proportion (height divided by waist girth) and muscularity (arm girth divided by waist girth) had significant protective effects against tie dissolution (estimate 0.187, *p*-value 0.032; estimate 0.175, *p*-value 0.033) while muscularity had no effect on tie formation. The results for body proportion are expected given that it is functionally and empirically correlated to BMI but as it is available at only a subset of exams (5–7) inferences are less precise than for BMI (Figure 3). Muscularity has a weaker association with BMI and so is a more unique measure.

There is a significant protective effect on tie dissolution if only the ego smokes (estimate −0.328, *p*-value 0.034) and a positive but not significant effect on tie formation if both the ego and the alter smoke (estimate 0.040, *p*-value 0.080). We conjecture that smokers, a minority and more and more socially outcast 10, have greater incentive to maintain friendships with non-smokers than vice versa. In the tie dissolution analysis, it was of interest to note that the effect of Smoke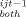 became even stronger when the dynamic reciprocity effects were added.

Depression score and blood pressure, the mutable but less observable traits, have no effect, although the null finding for depression is tempered by the fact that it was only measured consecutively in exams 5–7). Nonetheless, these results suggest that the observability of a health trait is an important determinant of its association with dyad status.

Height and personality type (A versus B), the two immutable and more observable traits, had no significant effects, suggesting that mutability might be another important characteristic of a health trait that affects dyad status. In results not reported, high average height appeared to be a barrier to tie formation.

Among the immutable and less observable traits, there were no significant effects. Thus, in general, there is no evidence that such traits influence the network dynamics. However, the effect of onlychild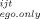 on dyad status has the opposite sign in the tie dissolution and tie-formation models (this is another case where significance is perhaps best portrayed as a joint statement across the tie-dissolution and tie-formation analyses—see Figure 4).

The fact that handedness, birth order, height, personality type, depression score, and blood pressure all have null effects, whereas the mutable and more observable traits (BMI, body proportion, smoking and to a lesser degree muscularity) had some significant results suggests that, among health traits, homophily might only occur on those that are observable (especially if they have social stigma) and mutable. However, the fact that immutable personal characteristics, such as baseline age and gender, have very strong homophily effects (discussed in Section 3.1 and by others; e.g. 4) reaffirms that, in general, mutability is not a necessary requirement for homophily.

### 3.3. Sensitivity analyses

We repeated the analyses with predictors for the trait at both of the two most recent exams as predictors (i.e. augmenting models (1) and (2) with variables for the key predictors evaluated at exam *t*−2). In this model, the coefficient of the trait at exam *t*−1 corresponds to the effect of the change in the trait between the two most recent exams whereas the coefficient at exam *t*−2 is the effect of the doubly lagged variable. No significant results were obtained for the effect of change in the trait between exams *t*−2 and *t*−1 on dyadic status in exam *t*, a result that is consistent with the Markov assumption.

We also evaluated the incremental effects of the health traits by grouping them in various combinations and re-fitting the models. In general, there was a trend towards reduced significance as more effects are added (as noted in Section 2.2, the increased clarity about the effect of Smoke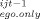 when the BMI variables are included in the model is an exception).

Finally, we tested whether the smoking variables interacted with time (represented by exam number). The rationale for this test is that the amount of critical comment and disparaging advertising about smoking has increased in recent years making the social stigma marking smoking ever the more isolating. We conjectured that homophily on smoking would increase over time. However, there was no apparent trend and the interaction was far from statistically significant.

## 4. Conclusion

When individuals with similar health risks are connected in a social network, it begs the question about whether such ties arose because of the shared traits or whether the shared traits arose because of the tie. Ideally, one would like to be able to perform some kind of randomized study to explore the questions considered in this paper. However, this would require randomly assigning individuals to dyads with different relationship states (unconnected, directional, or mutual) and experimentally perturbing their covariate values. The network would evolve over a period of time with data being collected on every individual and tie on the same regularly spaced dates. Such experiments are clearly not feasible in real-world settings. However, in this paper we have a rigorous series of analyses that attempt to approximate the underlying causal effects as closely and robustly as possible by utilizing the rich longitudinal nature of the FHS-Net.

The key findings are that homophily in BMI and smoking occurs with respect to tie dissolution and, to a lesser extent, tie formation. In addition, there is evidence of homophily in measures related to BMI, such as body proportion and muscularity, but as these were not measured at every exam the pool of evidence is smaller. Increasing BMI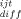 correlated with existing outbound ties dissolving, whereas decreasing BMI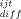 encourages the formation of new ties. With respect to smoking, if only the ego smokes, an existing tie was less likely to dissolve, and, if both individuals smoke, a new tie was more likely to form. We emphasize that we did not *ex ante* expect that a factor which causes one to form a friendship should weigh equally in decisions to end it. For instance, it may be that one does not realize, for instance, how troublesome it is to have a friend who is a smoker (for instance!) and so is quite happy to befriend people without reference to this attribute, but then is indeed affected when one is (on balance) considering whether a friendship is worth continuing or ending. Or perhaps people spend less time with their friends who are heavier or a smoker, hence having less time to bind the friendship together.

Homophily was not evident for depression, blood pressure, height, personality type, birth order, or left-handedness suggesting that two important characteristics of a health trait associated with the evolution of a network are observability (i.e. the trait itself, and changes in it, are easily observable by others) and, to a lesser extent, mutability. To the extent that depression status was known among friends and potential friends, we might have expected more definitive results; thus, the null results may reflect that depression was not that observable among the Framingham community. If either characteristic is absent, then dyadic status is unlikely to be affected by the health traits (or change in the health traits) considered in this paper. The fact that homophily is evident for the mutable and highly observable BMI and smoking traits but that only weak evidence is present for the other traits suggests that, in general, people seek out individuals who have come to resemble them or dissolve ties with those who come not to resemble them in obvious, health-related ways. It is important to recognize that this conclusion is limited to health traits since, clearly, age and gender homophily have strong effects on tie dissolution and tie formation and there is some evidence that being an only child has an effect on tie formation. Controlling for the lag of the geographic distance between individuals' abodes (weak evidence of homophily) and education (no evidence of homophily) does not alter these conclusions.

As expected, reciprocity had a protective effect against tie dissolution but unexpectedly was a discouragement to tie formation. As explained in Section 3.1, we attribute the latter result to the fact that most participants named a single close friend at each exam and a potential association between popularity and reciprocity due to the most popular individuals having very stable friendships and so being less likely to change their own friendship nomination.

There are several directions in which this work can be extended. We are currently working on developing a general longitudinal p2 model and associated software that can feasibly be implemented on large networks such as the FHS-Net. Methodological extensions beyond dyadic independence to allow transitivity and other higher order effects that account for clustering and other network phenomena are also possible. Because dyadic independence is in reality likely to be the exception as opposed to the norm and failing to account for strong triadic or higher order effects may lead to erroneous inferences 21, development of models that account for dependence structures among three or more actors and moreover of methods for fitting such models is important. In terms of accounting for cross-sectional dependence between dyads, the actor-oriented approach in SIENA is a leading candidate. However, in our experience, SIENA 28 only runs/yields results for networks with up to a few hundred actors, and not thousands of actors. Extensions of the exponential random graph 29 and the latent space 30 models have been proposed 31–33 but are also not used here because the size of the FHS-Net exceeds the capability of currently available software. Among these approaches, we view a generalization of latent-space models to longitudinal data as the most amenable to large networks. Furthermore, the scarcity of cross-sectional ties limits the utility of models that incorporate higher order dependence for analyzing the FHS-Net. Rather than compromise the data by using some *ad hoc* method to sample the network (there is no known method of sampling sociometric data such that all aspects of the structure of the network are preserved) to fit a more complex model, we used a model that could be fit to the whole FHS-Net (besides the random re-sampling of always null-dyads) in order to obtain useful results from this large and unique dataset.

In the future work, we also plan to develop multiple imputation methods for network data to avert the loss of information when multiple traits are included in a single model. Of particular interest is whether the use of network predictors (e.g. variables that summarize the extent of a trait in one's alters) in the imputation model yield imputed data that captures the structure of the complete network more accurately than imputations that ignore these predictors. In terms of our current analysis, influence from unobserved ties and actors that we do not capture will surely bias the results towards the null (not finding an effect) and so our results may err on the side of being too conservative.

A model that simultaneously models the effect of health traits and other individual characteristics on the ties in a network and also the effect of the network on health traits is the ultimate destination of longitudinal network analysis. Joint modeling ensures that the direction of causality is respected and obtains the most efficient inferences about the parameters, and if the model is correctly specified, also accounts for unobserved confounding. If it could be fit, such a model would surpass the current model in which the actor-specific random effects control for unmeasured time-invariant effects when the model is correctly specified but offers no safeguard against unmeasured time-varying confounders. As more and more data become available regarding human interactions observed in such a fashion that longitudinal information about both individual attributes and network topology is known (e.g. from passive/massive data collection efforts such as those involving cell phone networks, etc. 34), joint modeling will come to be a new frontier in studies of social networks.
